# Detection of Langat virus by TaqMan real-time one-step qRT-PCR method

**DOI:** 10.1038/srep14007

**Published:** 2015-09-11

**Authors:** Siti Fatimah Muhd Radzi, Claudia Rückert, Sing-Sin Sam, Boon-Teong Teoh, Pui-Fong Jee, Wai-Hong Phoon, Sazaly Abubakar, Keivan Zandi

**Affiliations:** 1 Tropical Infectious Disease Research Center (TIDREC), Department of Medical Microbiology, Faculty of Medicine, University of Malaya, 50603 Kuala Lumpur, Malaysia; 2 The Pirbright Institute, Ash Road, Pirbright, Surrey GU24 0NF, United Kingdom

## Abstract

Langat virus (LGTV), one of the members of the tick-borne encephalitis virus (TBEV) complex, was firstly isolated from Ixodes granulatus ticks in Malaysia. However, the prevalence of LGTV in ticks in the region remains unknown. Surveillance for LGTV is therefore important and thus a tool for specific detection of LGTV is needed. In the present study, we developed a real-time quantitative reverse-transcription-polymerase chain reaction (qRT-PCR) for rapid detection of LGTV. Our findings showed that the developed qRT-PCR could detect LGTV at a titre as low as 0.1 FFU/ml. The detection limit of the qRT-PCR assay at 95% probability was 0.28 FFU/ml as determined by probit analysis (*p* ≤ 0.05). Besides, the designed primers and probe did not amplify ORF of the E genes for some closely related and more pathogenic viruses including TBEV, Louping ill virus, Omsk hemorrhagic fever virus (OHFV), Alkhurma virus (ALKV), Kyasanur Forest Disease virus (KFDV) and Powassan virus (POWV) which showed the acceptable specificity of the developed assay. The sensitivity of the developed method also has been confirmed by determining the LGTV in infected tick cell line as well as LGTV- spiked tick tissues.

Langat virus (LGTV) is a member of the tick-borne encephalitis virus (TBEV) complex, which belongs to the genus flavivirus of the family *Flaviviridae*. Similar to other flaviviruses, LGTV is a lipid-enveloped virus carrying a positive-sense single-stranded RNA genome of approximately 11 kb in length. The LGTV RNA genome encodes a polyprotein, which has 84% amino acid homology with some strains of TBEV. The polyprotein is co- and post-translationally processed by the host and viral proteases into three structural [capsid (C), precursor membrane (preM) and envelope (E)] and seven non-structural (NS) proteins including NS1, NS2A, NS2B, NS3, NS4A, NS4B and NS5^1^.

LGTV was initially isolated from hard ticks of the species *Ixodes granulatus* in Malaysia^2^ and subsequently from *Haemaphysalis papuana* in Thailand^3^. Antibodies against LGTV have been detected in forest ground rats in Malaysia^2^, indicating the possibility of virus transmission through infected tick bite. While LGTV showed neurovirulence in laboratory mouse^4^,^5^ and hamster^6^,^7^ models, the virus has not been associated with any human disease under natural conditions. Nevertheless, the occurrence of neurological complications with long-term sequelae was reported in a very small percentage of individuals injected with live LGTV during vaccine and cancer treatment trials^8^,^9^, indicating the potential for virulence of LGTV in humans upon infection.

It has been shown that infection with LGTV elicited cross-reactive antibodies against other antigenically related flaviviruses of the TBEV complex^10^,^11^,^12^. Using the ELISA test, we recently found a high seroprevalence of TBEV IgG among aboriginal population of Peninsular Malaysia, Malaysia (authors’ unpublished data). While autochthonous TBEV infection has never been reported in Malaysia, LGTV is likely to be one of the main causative agents contributing to the seropositivity in the aborigines, as there is more than 80% of homology between the genome of TBEV and LGTV. The high percentage of antigenic similarity between TBEV and LGTV was the main reason that some investigators have studied LGTV as a potential candidate for vaccine development against TBEV. However, the current LGTV prevalence in ticks and humans in Malaysia is unknown. This suggests the importance of surveillance for the virus in the region and thus the need of a tool for specific detection of LGTV.

In the present study, we have designed and developed a TaqMan real-time quantitative reverse-transcription-polymerase chain reaction (qRT-PCR) method for rapid detection of LGTV.

## Results

### Virus propagation and titration in Vero cells

The LGTV infectious titre was obtained by calculating the numbers of visible foci from focus forming assay (FFA) 3 days post infection (poi) in Vero cells. In this study, the LGTV infectious titre was 1.45 × 10^6^ FFU/ml. The titre of this virus was used in determining the highest amount of LGTV RNA in the qRT-PCR which was 1.0 × 10^6^ FFU/ml.

### Generation of the clones for E gene of closely related viruses to LGTV

The synthesized sequences for the E gene of Louping ill virus, OHFV, ALKV, KFDV, and POWV as the closely related viruses to LGTV were cloned in pET-51b(+) plasmid. The data from sequencing of the constructed clones have shown that all E genes were cloned successfully.

### Generation of TBEV RNA

**Figure 1:**
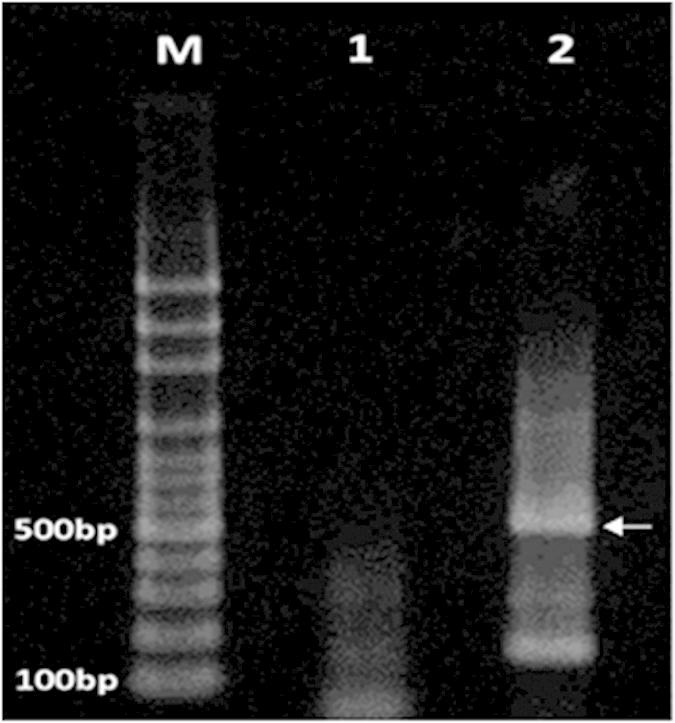
Confirmation of RT-PCR amplification for TBEV RNA. Lane M, 100 bp plus ladder; lane 1, negative control; lane 2, TBEV RNA. A RT-PCR product of the expected size (532 bp) is visible in lane 2.

For further assessment of specificity for the developed qRT-PCR, against the TBEV as the closest virus with LGTV, a TBEV RNA was used and generated via *in vitro* transcription of pTNd/5′ containing E gene of TBEV. The *in vitro* transcription yielded TBEV RNA at a concentration of 187 ng/μl. Amplification of a 532 bp DNA fragment by the TBEV RT-PCR assay indicated the presence of TBEV RNA (Fig. 1). The *in vitro*-transcribed TBEV RNA was used in the qRT-PCR for specificity evaluation.

### LGTV qRT-PCR assay

**Figure 2:**
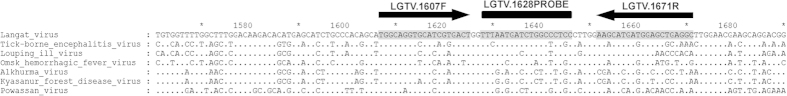
Map of LGTV-specific qRT-PCR primers and TaqMan probe in alignment with the viral E gene sequences of the LGTV related flaviviruses. The ORF for the E gene of Louping ill virus, Omsk hemorrhagic fever virus, Alkhumra virus, Kyasanur Forest disease virus, and Powassan virus have been aligned with the related ORF in LGTV genome using Clustal X 2.0 software. The arrows indicate the orientation of primers in 5′ to 3′ direction.

**Figure 3:**
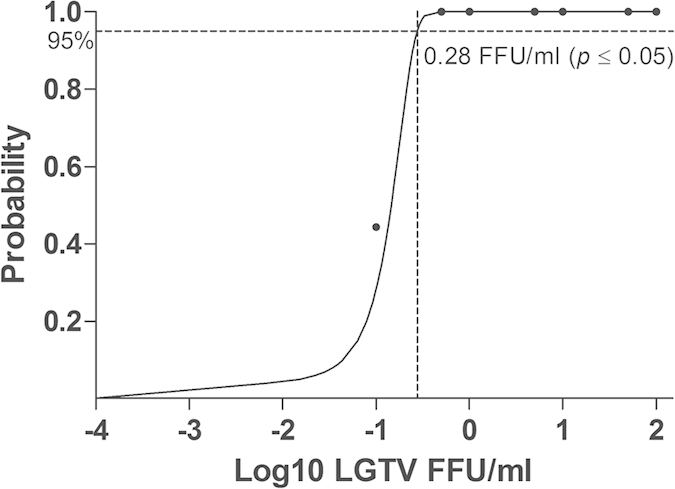
Detection limit of the qRT-PCR for detection of LGTV in nuclease-free water. The probit regression curve was obtained from nine replicates of LGTV RNA at seven dilutions (100, 50, 10, 5, 1, 0.5 and 0.1 FFU/ml).

**Figure 4:**
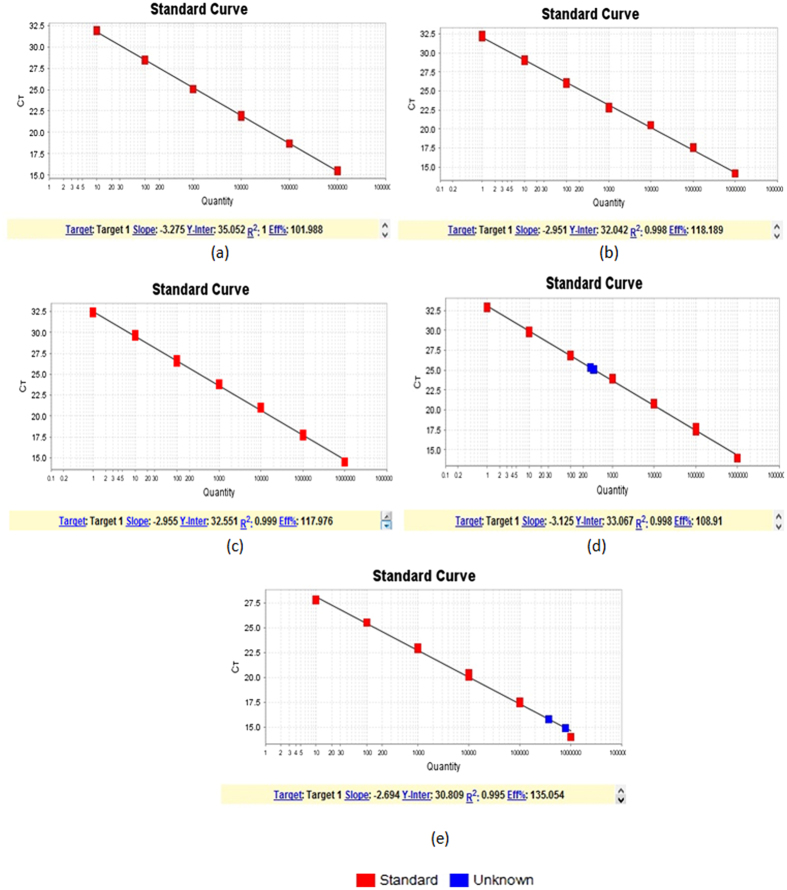
Standard curves for qRT-PCRs. (**a**) Standard curve of qRT-PCR for detection of LGTV. The amplification efficiency (Eff%) was 101.988 and coefficient of determination (R^2^) was 1. (**b**) Standard curve for specificity of LGTV qRT-PCR against five closely related viruses. The Eff% of qRT-PCR was 118.189 and the R^2^ was 0.998. The LGTV qRT-PCR assay didn’t amplify any of five closely related viruses while the negative control of the qRT-PCR was undetermined. (**c**) Standard curve for specificity of LGTV qRT-PCR against *in vitro* transcribed TBEV RNA. The Eff% of qRT-PCR was 117.976 and the R^2^ was 0.999. There was no *in vitro* transcribed TBEV RNA detected. (**d**) Standard curve of qRT-PCR for detection of LGTV RNA in spiked homogenized tick tissues. The Eff% of qRT-PCR was 108.91and the R^2^ was 0.998. LGTV RNA detected in spiked sample with LGTV at 212.24 FFU/ml and the other sample was spiked with TBEV and LGTV together at 239.11 FFU/ml (Blue boxes). (**e**) Standard curve of qRT-PCR for detection of LGTV RNA in IDE8 cells. The Eff% of qRT-PCR was 135.054 and the R^2^ was 0.995. LGTV RNA in IDE8 cells was detected at 406,268 FFU/ml (2 days poi) and 776,412 FFU/ml (4 days poi) (Blue boxes).

Two LGTV-specific primers and one TaqMan probe were designed for the detection and quantification of LGTV RNA. The forward primer sequence was 5′-TGGCAGGTGCATCGTGACT-3′, reverse primer sequence was 5′-GCCTCAGCTCCATCATGCTT-3′ and the TaqMan probe was: FAM-TTTAATGATCTGGCCCTCC-NFQ-MGB (Fig. 2). The detection limit of qRT-PCR assay at 95% probability was 0.28 FFU/ml (probit analysis, p ≤ 0.05) (Fig. 3).The developed qRT-PCR assay showed an amplification efficiency (Eff%)of 101.988 and a coefficient of determination (R^2^) of 1 for the detection of LGTV RNA while the negative control was undetermined (Fig. 4a).

### Specificity of the LGTV qRT-PCR assay

Initially, the similarity between the designed primers/probe and the ORF for E gene of LGTV and other related viruses have been calculated theoretically. Multiple sequence alignment of the viral E gene sequences has been done using Clustal X 2.0 software and as it is shown in Table 1 the most significant similarity has been found between the designed primers/probe and E gene ORF for LGTV with 100% similarity. Whereas the similarity between the sequences of designed primers/probe with TBEV and other related viruses were not too significant (Table 1). Therefore, the chance of detection of each related viruses with synthesized primers and probe was not considerable theoretically. However, the obtained data in this part of the study have been confirmed using synthesized E genes of all related viruses.

The constructed clones for all synthesized E genes of related viruses in pET-51b(+)vector have been evaluated by our developed LGTV qRT-PCR assay and as it is shown in Fig. 4b, there was no amplification for all those viruses with Eff % = 118.189 and R^2^ = 0.998.

Nevertheless, the specificity of LGTV qRT-PCR also determined against *in vitro*-transcribed TBEV RNA. The Eff% of LGTV qRT-PCR against TBEV was 117.976 and R^2^ was 0.999. There was no amplification for *in vitro*-transcribed TBEV RNA (Fig. 4c).

### Sensitivity of the LGTV qRT-PCR assay

To determine the sensitivity of the LGTV qRT-PCR assay, LGTV spiked crushed homogenized tick tissues and also LGTV infected IDE8 cells have been studied in two independent experiments. A homogenate of non-infected crushed tick tissue has been spiked with LGTV together with necessary controls including another crushed tick tissue sample, which has been spiked by LGTV and TBEV RNA as well as non-infected crushed tick tissue sample. As it is shown in Fig. 4d, our developed qRT-PCR method can detect LGTV but not TBEV RNA in both spiked samples. The Eff% of qRT-PCR was 108.91 and R^2^ was 0.998.

As a further investigation to prove the sensitivity of our developed qRT-PCR, extracted RNA of LGTV from the LGTV-infected IDE8 cell line has been evaluated in 2 and 4 days poi. The amplification of the LGTV RNA in LGTV-infected IDE8 cell line has been shown successfully with increasing RNA level through the day 2 to 4 poi that were equivalent to the 406,268 FFU/ml of LGTV in day 2 post infection and 776,412 FFU/ml in day 4 post infection. The Eff% of LGTV qRT-PCR was 135.054 and the R^2^ was 0.995. There was no amplification detected in the non-infected IDE8 cells as negative control for this assay (Fig. 4e).

## Discussion

In the present study, a novel real-time qRT-PCR assay was developed for detection of LGTV TP21 strain. We demonstrated that the qRT-PCR assay could detect the LGTV at a titer as low as 0.1 FFU/ml. Probit analysis determined that the 95% detection limit of the qRT-PCR assay was 0.28 FFU/ml. This showed that the developed qRT-PCR assay was sensitive. Besides, the sensitivity of the qRT-PCR was determined against viral spiked crushed homogenised tick tissues and viral infected IDE8 tick cell line with LGTV and LGTV/TBEV together. The purpose of spiking both LGTV and TBEV together was to determine whether the designed and developed qRT-PCR in this study could differentiate the LGTV from TBEV as the closest virus in terms of genome homology in infected tick tissues and cell line or not. However, our data have proven that the developed qRT-PCR only can detect and amplify the LGTV RNA in both LGTV spiked samples specifically. The negative controls including non-spiked tick tissues and TBEV-RNA spiked tick tissues showed no amplification. This showed the sensitiveness of our developed qRT-PCR.

Our data from LGTV-infected IDE8 cell line has shown that the LGTV RNA level has increased through the incubation time poi. This finding showed the capability of our developed method for quantification of LGTV in samples and also it has been proven that IDE8 cell line could be a proper tick cell line for LGTV replication and propagation. Interestingly, there was no amplification for non- LGTV infected IDE8 cells/crushed tick tissues as another evidence to prove the specificity of the developed qRT-PCR in this study.

In order to assess the specificity of the developed LGTV qRT-PCR assay in this study, the E gene for five closely related viruses including Louping ill virus, OHFV, ALKV, KFDV, and POWV were synthesized and cloned in pET-51b(+) vector followed by evaluation by developed LGTV qRT-PCR. We have shown that our developed qRT-PCR did not amplify the E gene of six closely related viruses despite of the genomic homology between all those viruses and LGTV that could be a proof for the specificity of the developed qRT-PCR. However, as one of the limitations of our study we did not have access to all those viruses in our lab.

Furthermore *in vitro*-transcribed TBEV RNA was used to evaluate specificity of LGTV qRT-PCR. From Fig. 4c there was no amplification of the *in vitro*-transcribed TBEV RNA. This showed that the developed qRT-PCR can detect LGTV RNA only. The percentage of identity of LGTV primers and probe against closely related viruses in Table 1 showed the specificity of the developed qRT-PCR.

Campbell and colleagues previously developed a RT-PCR assay for detection of LGTV using a primer set targeting the viral C-prM region. They tested the RT-PCR on LGTV-infected Vero cell tissue culture supernatant fluid. The RT-PCR primers however showed cross-reaction with TBEV^9^.In the present study, we developed a real-time qRT-PCR assay for specific detection of LGTV and unlike to previous study, the qRT-PCR primers and TaqMan probe designed in our study targeted regions of the viral E gene that are conserved among LGTV but vary from those of closely related TBEV. In addition, the qRT-PCR assay can be completed in a shorter time (approximately 40 min) than the RT-PCR as post-amplification analysis by gel electrophoresis is not required.

As one of the limitations for our study we did not use viable intact TBEV and other related viruses due to unavailability of those viruses in our laboratory and also our collaborator’s laboratory. However, all mentioned viruses are categorized under the Biosafety Level 3 (BSL3) pathogens in Malaysia^13^ as they are not prevalent in the region. Nevertheless, we have tried to evaluate the specificity of our developed method against synthesized E genes of all related viruses as an alternative solution following theoretical study by alignment of primers/probe with relative viral ORFs. Therefore, we suggest the further evaluation of our developed method using all related infectious viruses for the future studies. Nevertheless, the developed qRT-PCR assay in this study would provide a good start for surveillance and exploration of new LGTV samples in the region.

In conclusion, the qRT-PCR assay developed in our study is sensitive and specific for the rapid detection of LGTV. While there is limited information describing LGTV infection in humans and ticks, the qRT-PCR assay could eventually help to improve virological surveillance in the regions where LGTV is endemic.

## Methods

### Cells

Vero cells (African green monkey kidney cells) were cultured in Eagle’s Minimum Essential Medium (EMEM) (Gibco, NY, USA) supplemented with 10% fetal bovine serum (FBS) (Gibco, NY, USA), 2 mM L-glutamine (Gibco, NY, USA) and 0.1 mM non-essential amino acids (Sigma-Aldrich). Vero cells were maintained at 37 °C in an atmosphere of 5% CO_2_in air.

IDE8 cells (derived from *Ixodes scapularis* embryos) were provided from The Tick Cell Biobank, Pirbright Institute, United Kingdom. IDE8 cells were cultured in Liebovitz’s L15 medium supplemented with 5% FBS, 10% Tryptose Phosphate Broth, 0.1% Bovine Lipoprotein Cholesterol, 1% L-glutamine and 1% Fungizone/ Penicilin/ Streptomycin. The cells were maintained at 32 °C.

### Virus

The LGTV TP21 strain used in this study was kindly provided by Dr. Sonja Best of the National Institute of Allergy and Infectious Diseases (NIAID), National Institutes of Health (NIH), USA and Professor John Fazakerley from Pirbright University, UK. LGTV was propagated by inoculating monolayers of Vero cells in 75 cm^2^ cell culture flasks at ~80% confluency with initial virus inoculum diluted 1:200 in EMEM supplemented with 2% FBS (corresponding to approximately 10^4^ FFU/ml). After 1 h of adsorption at room temperature with gentle rocking, the cells were cultured in EMEM supplemented with 2% FBS and incubated as above. Virus-containing cell culture supernatant was harvested on day sevenpost-infection (poi) after observation of cytopathic effects. Harvested supernatant was sterile-filtered, aliquotted and stored at −80 °C until further use.

### Virus titration

Viral FFA using Vero cells determined virus infectivity titre of the LGTV. Cells were seeded at 6 × 10^5^ cells/well in 24-well plates and cultured overnight as above. Growth medium was removed and 200 μl of 10-fold serially diluted LGTV(1:10 to 1:10^9^) in EMEM with 2% FBS was added to the wells. After 1 h of adsorption at 37 °C in the presence of 5% CO_2_, the medium was replaced with 500 μl of overlay comprising EMEM containing 2% FBS and 1.5% carboxymethylcellulose (Sigma-Aldrich, USA). The cells were incubated for 3 days as above. After 3 days of incubation, foci of infected cells were detected by a peroxidase-based viral focus-staining assay. In brief, the overlay was discarded and cells were washed gently three times with phosphate buffered saline (PBS). The cells were fixed with 4% paraformaldehyde for 30 min at room temperature followed by three washes with PBS. 300 μl of 1% IgepalCA-630 detergent (Sigma-Aldrich, St. Louis, MO, USA) was added to permeabilize the cells for 15 min at room temperature. Subsequently, the cells were washed three times with PBS and blocked with 3% skimmed milk (Sigma, St. Louis, MO, USA) in PBS for 2 h at room temperature. After another three washes with PBS, the cells were incubated with anti-flavivirus monoclonal antibody D14G2 (Merck Millipore) diluted 1:500 in 1% skimmed milk in PBS at 37 °C for 1 h. Cells were then washed three times with PBS and incubated with goat-anti-mouse IgG conjugated with horse-radish peroxidase (Merck Millipore) at a dilution of 1:250 in 1% skimmed milk in PBS for 1 h and 10 min at 37 °C. After washing three times with PBS, Metal-Enhanced DAB Substrate (Thermo Scientific Pierce, Rockford, IL, USA) was added to each well for 10 min to stain the virus foci. Foci were counted under a SMZ 1000 stereomicroscope (Nikon, Tokyo, Japan) and the virus titre was expressed as focus-forming units/ml (FFU/ml).

### LGTV RNA extraction

LGTV RNA was extracted from virus stock using the QIAmp Viral RNA Mini Kit (Qiagen, Hilden, Germany), following the manufacturer’s instructions and extracted RNA stored at −80 °C until further use.

### Synthesizing of E genes for LGTV-related viruses

The envelope gene of five closely related viruses: Louping ill virus, OHFV, ALKV, KFDV, and POWV were used to determine the specificity of the LGTV qRT-PCR assay. The sequences for all viral E genes used in this study were retrieved from GenBank (Table 2). The E genes of the five viruses were synthesized and cloned in pET-51b(+) vector by Genscript company (Genscript, Piscataway, NJ) followed by sequencing to show the accuracy of the gene synthesis.

### Generation of TBEV RNA

The plasmid (pTNd/5′) containing cDNA derived from TBEV strain Neudoerfl was kindly provided by Professor Franz Heinz of the Department of Virology, Medical University of Vienna, Austria and used in this study to assess the specificity of the qRT-PCR assay for LGTV detection. The pTNd/5′ was transformed into NovaBlue competent cells (Novagen Inc. USA) for propagation. After overnight bacterial culture, the bacterial cells were lysed and the pTNd/5′ was extracted using a Qiagen Plasmid Extraction kit (Qiagen, Hilden, Germany).

The pTNd/5′ was linearized by digestion with NheI-HF (New England Biolabs, USA) at 37 °C for 1 h and 65 °C for 20 min. The digested pTNd/5′was analyzed by agarose gel electrophoresis. The linearized pTNd/5′ was extracted and purified from the agarose gel using a QiaQuick Gel Extraction kit (Qiagen, Hilden, Germany) according to the manufacturer’s protocol. Subsequently, the purified pTNd/5′was transcribed by T7 RNA polymerase using a MEGAscript® T7 Kit (Ambion, Austin, TX, USA) according to the manufacturer’s instructions. Briefly, 1 μg of purified pTNd/5′ was added to a 20 μl transcription reaction containing 2 μl of each of ribonucleotide solutions (ATP, CTP, GTP, and UTP), 2 μl of 10 × reaction buffer and 2 μl of T7 RNA Polymerase enzyme mix. The reaction was incubated at 37 °C for 4 h. After *in vitro* transcription, the transcription product was treated with 1.0 μl DNase and the concentration of RNA was measured using a NanoPhotometer (Implen).

The RT-PCR was performed to amplify the TBEV RNA using the Access RT-PCR System (Promega, USA). The reaction was prepared in 25 μl of RT-PCR mixture consisting of 15.5 μl nuclease-free water, 5.0 μl 1 × AMV/Tfl buffer, 0.5 μl of 0.2 mMdNTP mix, 1.0 μl of 1 mM MgSO_4_, 0.5 μl of 0.1 u/μl AMV RT, 0.5 μl of 0.1 u/μl Tfl DNA Polymerase, 0.5 μl of 1 μM of each primer (Forward primer: 5′-ATGGCACCTGTGTGATCCTG-3′, Reverse primer: 5′-ATTGAAGGCTTCCCCTCAGC-3′) and 1.0 μl of TBEV RNA template. The RT-PCR was performed at the following amplification cycles: reverse transcription at 45 °C for 45 min, initial denaturation at 94 °C for 2 min, followed by 40 cycles of 94 °C for 30 s, 60 °C for 1 min and 68 °C for 2 min. The amplified product was analyzed by electrophoresis using 1.5% agarose gel. The gel was stained with GelRed (Biotium Inc., US)and the amplicon was visualized under UV light.

### Preparation of LGTV spiked tick lysate

Ticks were collected from Kampung Tumboh Hangat and Kampung Sungai Perah, Perak. The ticks were stored in 70% ethanol for 3 min, 5% bleached for 3 min and washed twice with double distilled water for 3 min followed by drying on filter paper for 3 min. In next step, the sample was frozen in liquid nitrogen and powdered with a steel mortar and pestle followed by homogenizing the lysate in PBS. Following a brief spin, the homogenized tick tissue sample was spiked with 10^3^ FFU/ml of LGTV and one sample spiked with LGTV and TBEV. The negative controls consisted of non-spiked tick tissues and also one sample with TBEV- spiked tick tissue. The RNA was extracted and stored at −80 °C until further use.

### Preparation of LGTV infected IDE8 cells

The sensitivity of LGTV qRT-PCR was evaluated via LGTV RNA extracted from LGTV infected-IDE8 cells as well. IDE8 cells were seeded at 1 × 10^6^ cells/tube in flat-sided tube and cultured overnight as above. Growth medium was removed and 200 μl of LGTV in L15 medium with 2% FBS was added into the tube. Cells in negative control tube were maintained with medium with no LGTV. After 1 h of adsorption at 32 °C, the cells were washed gently three times with serum free L15 medium and replaced with 3 ml of L15 medium with 2% FBS. The cells were incubated for 2 days and 4 days at temperature as above. After 2 days and 4 days of incubation, RNA of LGTV was extracted from IDE8 cells using the RNeasy® Plus Mini Kit (Qiagen, Hilden, Germany), following the manufacturer’s instructions. All RNA extraction was performed manually. The RNA was eluted in 40 μl of RNase-free water and stored at −80 °C until further use.

### Design of LGTV-specific qRT-PCR primers and probe

The LGTV-specific primers and TaqMan probe used for the qRT-PCR assay were designed based on conserved regions of the E gene of LGTV. Sequences of the E genes from the closely related viruses TBEV, Louping ill virus, OHFV, ALKV, KFDV, and POWV^14^ (Table 2) were used during the design process to avoid cross-reactivity of the LGTV primers and probe. All the viral E gene sequences used in this study were retrieved from GenBank. Multiple sequence alignment of the viral E gene sequences was performed using Clustal X 2.0^15^.

### Primers and probe analysis relative to LGTV E gene ORF

The E gene sequence of LGTV and closely related viruses were retrieved from GenBank (Table 2). Forward primer, reverse primer and TaqMan probe of LGTV qRT-PCR was analysed relative to LGTV E gene ORF at position of forward primer: 1607–1625; reverse primer: 1652–1671 and probe: 1628–1646. Percentage of identity was calculated to determine the specificity of the LGTV qRT-PCR primers and probe against closely related viruses.

### Development of qRT-PCR assay

The qRT-PCR was performed in a total reaction volume of 12.0 μl consisting of1 × TaqMan® Fast Virus 1-step Master Mix (Applied Biosystems, USA), 1 × CustomTaqMan® Gene Expression Assays (Applied Biosystems, USA), and 2 μl of RNA template. The qRT-PCR standard curve, ranging from 1 to 10^6^ FFU/ml, was generated from a 10-fold serial dilution of LGTV RNA extracted from the virus stock with known viral titer. All amplifications were performed in triplicate using the StepOnePlus™ Real-Time PCR System (Applied Biosystems, USA) with the following amplification cycles: reverse transcription at 50 °C for 5 min, initial denaturation at 95 °C for 20 s, followed by 40 cycles of 95 °C for 3 s and 60 °C for 30 s. Raw data was analyzed with StepOne Software v2.2.1 to determine the amount of viral RNA based on the threshold cycles (Ct). The efficiency of the qRT- PCR was measured from the slope of the standard curve.

### Detection limit of developed qRT-PCR assay

The detection limit of the qRT-PCR assay was assessed by using a panel of serially diluted viral RNA samples in nuclease-free water extracted from culture supernatant with known viral titers of 100, 50, 10, 5, 1, 0.5 and 0.1 FFU/ml (quantitated by viral titration assay). The qRT-PCR detection limit test was repeated nine times. A probit analysis was performed using IBM SPSS Statistics, version 21 (IBM Corporation, New York, United States) to calculate the detection limit of the qRT-PCR assay at 95% probability.

## Additional Information

**How to cite this article**: Muhd Radzi, S. F. *et al*. Detection of Langat virus by TaqMan real-time one-step qRT-PCR method. *Sci. Rep*. **5**, 14007; doi: 10.1038/srep14007 (2015).

## Figures and Tables

**Table 1:** Percentage of identity of LGTV primers and probe relative to ORF of the E gene of LGTV and closely related viruses.

Virus	Target gene	Position 1607–1625 relative to Langat ORF (forward primer) 5′-TGGCAGGTGCATCGTGACT-3′		Position 1652–1671 relative to Langat ORF (reverse primer) 5′-GCCTCAGCTCCATCATGCTT-3′		Position 1628–1646 relative to Langat ORF (probe) 5′-TTTAATGATCTGGCCCTCC-3′	
LGTV	E	TGGCAGGTGCATCGTGACT	100% identical	AAGCATGATGGAGCTGAGGC	100% identical	TTTAATGATCTGGCCCTCC	100% identical
TBEV	E	TGGCAGGT**C**CAT**A**GG**G**ACT	84.21% identical	AA**A**CATGA**G**GGAGC**GC**A**AAA**	65% identical	TT**C**AATGATCTGGC**T**CT**G**C	84.21% identical
Louping ill	E	TGGCAGGT**C**CA**C**CG**A**GACT	84.21% identical	AAGCATGATGGA**AACCCACA**	60% identical	TTTAATGA**C**CTGGC**T**CTCC	89.47% identical
OHFV	E	TGGCAGGT**T**CA**CA**G**A**GA**T**T	73.68% identical	AA**A**CATGA**G**GGA**ATG**G**T**GG**G**	65% identical	TTTAA**C**GATCT**A**GC**T**CT**G**C	78.95% identical
ALKV	E	TGGCAGGTGCA**C**CG**A**GACT	89.47% identical	**CGA**CA**C**GA**G**GG**C**GC**CC**A**T**G**A**	50% identical	TTT**G**A**A**GA**C**CT**CT**CC**T**T**G**C	63.16% identical
KFDV	E	TGGCAGGT**A**CA**C**CG**A**GACT	84.21% identical	**CGA**CA**C**G**GG**GG**T**GC**CC**AGG**A**	50% identical	TTT**G**A**G**GA**C**CT**CT**CC**T**T**G**C	63.16% identical
POWV	E	TGGCA**A**GTGCA**C**CGTGACT	89.47% identical	AA**A**CA**CA**A**G**G**ACAACC**A**A**G**A**	40% identical	TTT**G**A**G**GA**CT**TGGC**G**CT**G**C	68.42% identical

Nucleotides in Bold and Underlined Character are non-identical at positions relative to the LGTV E gene ORF. As it is summarized in the table the highest similarity is between TBEV and LGTV.

**Table 2:** List of flavivirus E gene sequences used for the design of the LGTV-specific qRT-PCR primers and probe with their respective accession numbers.

Virus	Accession number
Langat virus	NC_003690
Tick-borne encephalitis virus	NC_001672
Louping ill virus	NC_001809
Omsk hemorrhagic fever virus	NC_005062
Alkhumra virus	NC_004355
Kyasanur Forest disease virus	JF416960
Powassan virus	NC_003687
